# Mdivi-1 Protects CD4^+^ T Cells against Apoptosis via Balancing Mitochondrial Fusion-Fission and Preventing the Induction of Endoplasmic Reticulum Stress in Sepsis

**DOI:** 10.1155/2019/7329131

**Published:** 2019-05-16

**Authors:** You Wu, Yong-Ming Yao, He-Liang Ke, Lan Ying, Yao Wu, Guang-Ju Zhao, Zhong-Qiu Lu

**Affiliations:** ^1^Emergency Department, The First Affiliated Hospital of Wenzhou Medical University, Wenzhou, China; ^2^Wenzhou Municipal Key Laboratory of Emergency, Critical Care and Disaster Medicine, The First Affiliated Hospital of Wenzhou Medical University, Wenzhou, China; ^3^Trauma Research Center, First Hospital Affiliated to the Chinese PLA General Hospital, Beijing, China

## Abstract

Apoptosis of CD4^+^ T cells plays a central role in the progression of sepsis because it is associated with subsequent immunosuppression and the lack of specific treatment. Thus, developing therapeutic strategies to attenuate the apoptosis of CD4^+^ T cells in sepsis is critical. Several studies have demonstrated that Mdivi-1, which is a selective inhibitor of the dynamin-related protein 1 (Drp1), attenuates apoptosis of myocardial cells and neurons during various pathologic states. The present study revealed the impact of Mdivi-1 on the apoptosis of CD4^+^ T cells in sepsis and the potential underlying mechanisms. We used lipopolysaccharide (LPS) stimulation and cecal ligation and puncture (CLP) surgery as sepsis models *in vitro* and *in vivo*, respectively. Our results showed that Mdivi-1 attenuated the apoptosis of CD4^+^ T cells both *in vitro* and *in vivo*. The potential mechanism underlying the protective effect of Mdivi-1 involved Mdivi-1 reestablishing mitochondrial fusion-fission balance in sepsis, as reflected by the expression of the mitofusin 2 (MFN2) and optic atrophy 1 (OPA1) , Drp1 translocation, and mitochondrial morphology, as observed by electron microscopy. Moreover, Mdivi-1 treatment reduced reactive oxygen species (ROS) production and prevented the induction of endoplasmic reticulum stress (ERS) and associated apoptosis. After using tunicamycin to activate ER stress, the protective effect of Mdivi-1 on CD4^+^ T cells was reversed. Our results suggested that Mdivi-1 ameliorated apoptosis in CD4^+^ T cells by reestablishing mitochondrial fusion-fission balance and preventing the induction of endoplasmic reticulum stress in experimental sepsis.

## 1. Introduction

Sepsis is a serious clinical complication, which results from the host's response to infection, traumatic injury, and surgery. Sepsis with subsequent life-threatening organ dysfunction syndrome is a major cause of death in intensive care units [1]. It has been reported that immunosuppression is considered to contribute to the late phase mortality of sepsis, which leads to uncontrolled primary infection and increases the susceptibility of the host to secondary infection [2, 3]. Immunosuppression is primarily caused by the apoptosis-induced loss of immune cells [4, 5], including dendritic cells, monocytes, CD4^+^ T and cells [6]. Particularly, apoptosis of CD4^+^ T cells plays a central role in the progression of immune suppression. Therefore, the present study focused on a novel therapeutic strategy that targeted apoptosis in CD4^+^ T cells to affect the outcome of septic patients.

Midvi-1 is a derivative of quinazolinone, which has been reported as an inhibitor of Drp1 function. Several studies have demonstrated that Mdivi-1 attenuates the apoptosis of myocardial cells and neurons during various pathologic states, such as Parkinson's disease, heart ischemia-reperfusion models, and Alzheimer's disease [7–10]. However, the effect of Mdivi-1 on the apoptosis of CD4^+^ T cells has not been elucidated so far. Herein, we investigated whether Mdivi-1 affects apoptosis of CD4^+^ T cells in sepsis and the potential underlying mechanisms.

A balance of mitochondrial fusion and fission is essential for maintaining mitochondrial functions, including mitochondrial DNA (mtDNA) replication, energy metabolism, and calcium storage [11, 12]. In the context of sepsis, the mitochondrial fusion-fission balance shifts toward mitochondrial fission, resulting in an exacerbation of ROS generation, an increase in mitochondrial outer membrane permeabilization and cytochrome *c* release [13]. Persistent high levels of ROS ultimately lead to cell apoptosis [14]. The processes of mitochondrial dynamics are regulated by conserved dynamin-related GTPases, including fission protein Drp1, its receptor Fis1, and the fusion proteins MFN2 and OPA1 [15]. During mitochondrial fission, Drp1 is recruited from the cytosol into the outer mitochondrial membrane and forms oligomers to divide a single mitochondrion into two separated mitochondria. Mdivi-1 is a selective Drp1 inhibitor that has been reported to reduce mitochondrial fission and ROS generation [13]. Mdivi-1 blocks mitochondrial fission via preventing Drp1 oligomerization, and this selective inhibition of mitochondrial fission suppresses mitochondrial fusion defects to recover mitochondrial fusion-fission balance [16, 17]. Therefore, Mdivi-1 likely restores mitochondrial fusion-fission balance in sepsis. Additionally, endoplasmic reticulum (ER) function was disrupted and ER stress (ERS) was induced in sepsis; then, cell apoptosis appears to be initiated in response to acute or chronic ER stress [18–20]. Several studies have shown that Mdivi-1 prevents the induction of ER stress in pathologic states [21–23].

**Figure 1:**
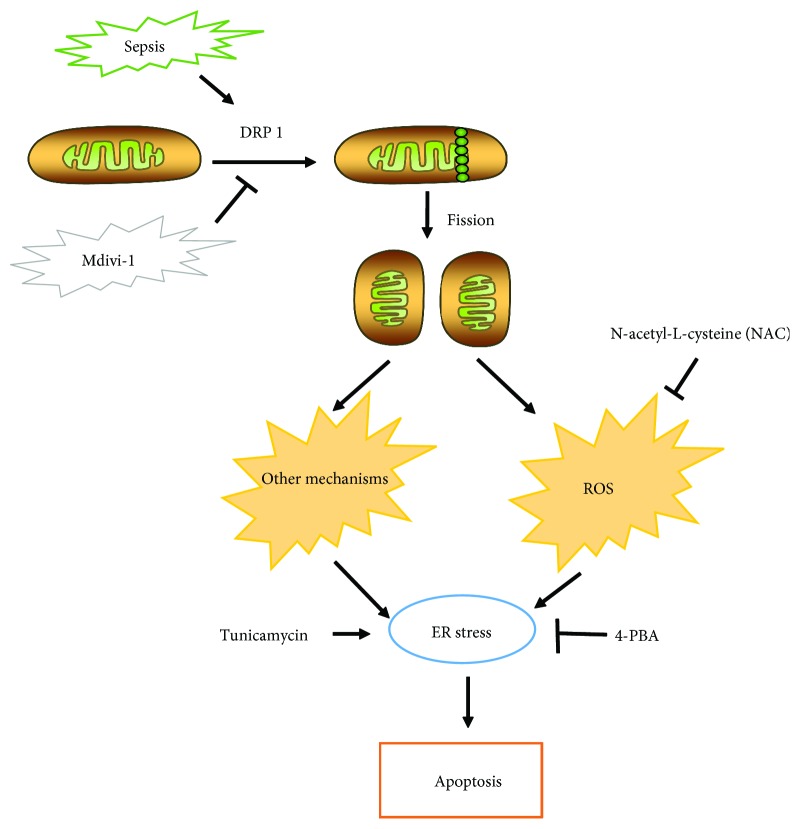
Schematic representation illustrating the hypothesis. During sepsis, Drp1 is recruited from the cytosol into the mitochondrial outer membrane and forms oligomers to divide a single mitochondrion into two separated mitochondria, which is termed mitochondrial fission. Enhanced mitochondrial fragmentation could lead to mitochondrial dysfunction, which induces ER stress. Interestingly, mitochondrial fission induces ER stress by, at least partly, overproduction of ROS. Therefore, we hypothesized that Mdivi-1, a selective inhibitor of Drp1, protects CD4^+^ T cells against apoptosis and is probably through reestablishing the mitochondrial fusion-fission balance and preventing the induction of ER stress. Moreover, ROS was a potential connection between mitochondrial dynamics and ER stress.

Herein, we aimed to verify the hypothesis that Mdivi-1 protected CD4^+^ T cells against apoptosis in sepsis. We found that Mdivi-1 protects CD4^+^ T cells against apoptosis likely through reestablishing the mitochondrial fusion-fission balance and preventing the induction of ER stress (Figure 1).

## 2. Materials and Methods

### 2.1. Animals and Surgical Procedures

Male BALB/c mice (6 to 8 weeks old and weighing 19 to 21 g) were purchased from the Laboratory Animal Institute, Chinese Academy of Medical Science, Beijing, China. All animals were housed under specific pathogen-free and temperature-controlled conditions. All animal experiments were performed in accordance with the National Institute of Health Guide for the Care and Use of Laboratory Animals with the approval of the Scientific Investigation Board of the Chinese PLA General Hospital, Beijing, China. All animals had free access to water but were fasted for 16 h before the surgical procedure. The mice were anesthetized with 5% chloral hydrate (300 mg/kg of body weight, Sigma, St. Louis, MO), and their lower abdominal regions were incised approximately 1 cm to expose the cecum. The cecum was ligated at the middle and then was punctured twice with a 21-gauge needle. The mice in the sham group underwent the same laparotomy procedure without the ligation and puncture. A subcutaneous injection of 1 mL of sterile saline was administered to the mice for fluid resuscitation after surgery. The mice were randomly divided into the following four groups: sham+DMSO, sham+Mdivi-1, CLP 24 h+DMSO, and CLP 24 h+Mdivi-1. Mdivi-1 (purchased from Sigma, St. Louis, MO), a selective mitochondrial division inhibitor, was injected at 20 mg/kg intraperitoneally 1 h prior to the surgery.

### 2.2. Cell Preparation and Treatment

The spleens were harvested from the mice and washed with ice-cold PBS, and then, the cells were separated through a 40 *μ*m mesh. Mononuclear cells were obtained by NycoPrep 1.007 A (Axis-Shield Co., Norway) density gradient centrifugation, and CD4^+^ T cells were then isolated from the mononuclear cells by negative selection using MicroBeads and a MiniMACS^TM^ Separator (Miltenyi Biotec, Bergisch Gladbach, Germany) according to the manufacturer's instructions. The isolated CD4^+^ T cells were incubated in RPMI 1640 FCS (10%) culture medium at 37°C in 5% CO_2_.

The cells were stimulated with 5 *μ*g/mL concanavalin A (ConA, Sigma, St. Louis, MO) for 24 h and then incubated with LPS (*Escherichia coli* strain, Sigma, St. Louis, MO) at a concentration of 100 ng/mL for 24 h. Then, the indicated concentration of Mdivi-1 (20 *μ*M) was added to the cells 30 min prior to the addition of LPS. Furthermore, the indicated concentration of tunicamycin (1.6 *μ*g/mL) or N-acetyl-L-cysteine/NAC (10 mM) or 4-phenylbutyric acid/4-PBA (2 mmol/L) was dissolved in the culture medium and added to the cells 30 min prior to the addition of LPS.

### 2.3. TUNEL Assay

**Figure 2:**
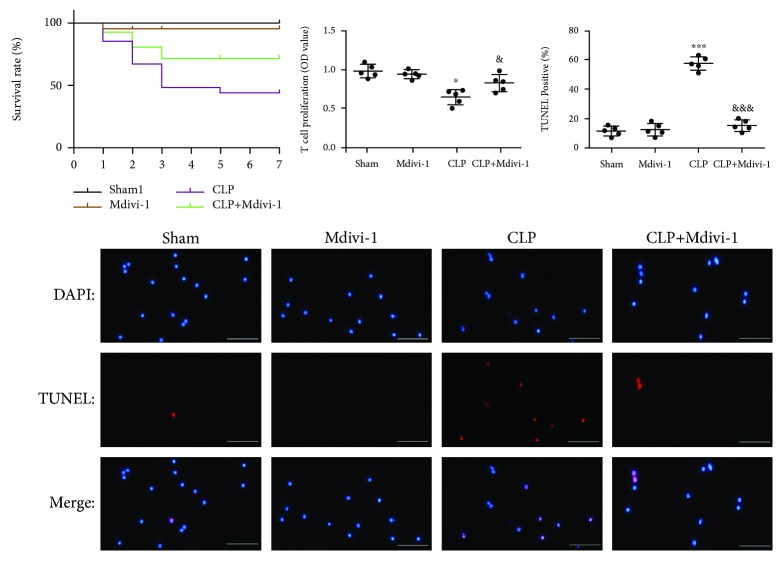
Mdivi-1 decreased apoptosis of CD4^+^ T cells after CLP. Mdivi-1 (20 mg/kg) was administered 1 h prior to the CLP procedure, and the mice were sacrificed at 24 h after the CLP procedure. (a) Survival rate of the mice over 7 days (*n* = 54). (b) Viability analysis of CD4^+^ T cells. (c) Representative photomicrographs of CD4^+^ T cells (around 20 cells) after colabeling TUNEL/DAPI and around 400 cells in each group evaluated to determine the percentage of TUNEL-positive cells. Scale bars, 50 *μ*m. The results are shown as the mean ± SD; *n* = 5. ^∗^ *P* < 0.05 vs. the sham group (^∗∗∗^ *P* < 0.001); ^&^ *P* < 0.05 vs. the CLP group (^&&&^ *P* < 0.001).

**Figure 3 fig3:**
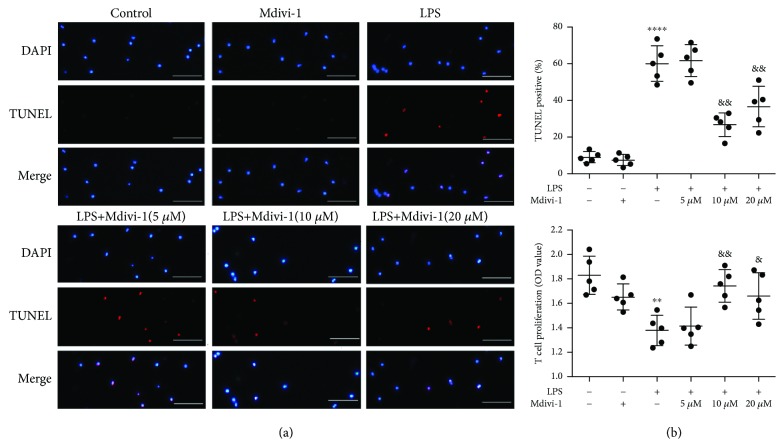
Mdivi-1 protects CD4^+^ T cells against LPS-induced apoptosis. Isolated CD4^+^ T cells were activated by ConA (5 *μ*g/mL), and then, Mdivi-1 (0, 10, and 20 *μ*M) was administered 30 min prior to the LPS stimulation. (a) Representative photomicrographs of CD4^+^ T cells (around 20 cells) after colabeling TUNEL/DAPI and around 400 cells in each group evaluated to determine the percentage of TUNEL-positive cells. (b) Viability analysis of CD4^+^ T cells. Scale bars, 50 *μ*m. The results are shown as the mean ± SD; *n* = 5. ^∗∗^ *P* < 0.01 vs. the control group (^∗∗∗∗^ *P* < 0.0001); ^&^ *P* < 0.05 vs. the LPS group (^&&^ *P* < 0.01, ^&&&^ *P* < 0.001).

**Figure 4:**
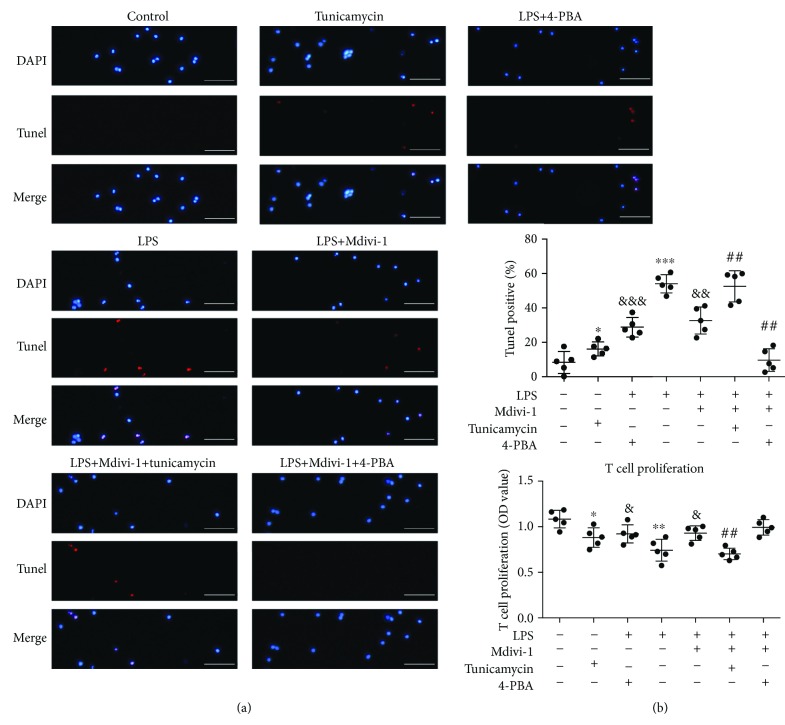
ERS induction compromised and ERS prevention amplified the protective effects of Mdivi-1 on CD4^+^ T cells after LPS treatment. Activated CD4^+^ T cells were copretreated with Mdivi-1 (20 *μ*M) and tunicamycin (1.6 *μ*g/mL) or 4-PBA (2 mmol/L) before LPS stimulation. (a) Representative photomicrographs of CD4^+^ T cells (around 20 cells) after colabeling TUNEL/DAPI and around 400 cells in each group evaluated to determine the percentage of TUNEL-positive cells. (b) Viability analysis of CD4^+^ T cells. Scale bars, 50 *μ*m. The results are shown as the mean ± SD; *n* = 5. ^∗^ *P* < 0.05 vs. the control group (^∗∗^ *P* < 0.01, ^∗∗∗^ *P* < 0.001); ^&^ *P* < 0.05 vs. the LPS group (^&&^ *P* < 0.01, ^&&&^ *P* < 0.001); ^##^ *P* < 0.01 vs. the LPS+Mdivi-1 group.

The apoptotic rate of the cells was measured using the One Step TUNEL assay (Beyotime Biotechnology, Shanghai, China). After pretreatment, the cells from each group were incubated with 50 *μ*L of TUNEL and incubation was darkly continued for 30 min. Then, the cell nuclei were stained with DAPI, and fluorescence microscopy was used to observe the cells. The total cell number of one sight (around 400 cells) was determined according to the DAPI stain, and the apoptotic cells were counted (defined by TUNEL-positive staining). The cell number of representative images in Figures 2 [Fig fig3]–4 is around 20.

### 2.4. T Cell Proliferation Assay

CD4^+^ T cells (5 × 10^4^ cells/well) were inoculated into a plate (96-well). After incubation with designated treatment, the proliferative rate of CD4^+^ T cells was assessed using a CCK-8 kit (Dojindo, Kumamoto, Japan) according to the manufacturer's instructions. CCk-8 (10 *μ*L/well) was added to each well, and incubation was continued for 1 to 4 h; then, the optical density was measured by a microplate reader (Spectra MR, Dynex, Richfield, MN) at a wavelength of 450 nm.

### 2.5. Preparation and Purification of the Mitochondrial Fractions

Mitochondrial proteins were collected using hypotonic disruption and differential centrifugation. Briefly, the harvested cells were suspended in cell lysis buffer (250 mM sucrose; 1 mM DTT; 10 mM KCl; 1 mM EDTA; 1 mM EGTA; 1.5 mM MgCl_2_; phenylmethylsulfonyl fluoride; 20 mM HEPES; pH 7.4; Applygen Technologies Inc., Beijing, China) at 4°C. The cell lysate was centrifuged at 800 ×*g* at 4°C for 10 min, and the supernatant was further centrifuged at 15000 ×*g* for 10 min. The supernatant contained the cytoplasmic fraction. The mitochondrial pellet was further resuspended in a mitochondrial lysis buffer, and then centrifugation at 24000 ×*g* was performed to obtain the mitochondrial fraction.

### 2.6. Western Blotting

The prepared proteins were boiled at 95°C for 3 min after mixing with SDS-loading buffer. Equal amounts of protein were separated using electrophorese on 8% to 10% polyacrylamide gel (Pulilai Co., Beijing, China) and transferred to nitrocellulose membranes. The membranes were blocked with 10% skimmed milk, and then, the membranes were incubated with following primary antibodies: anti-Drp1/anti-p-Drp1 (1 : 1000, #5391/4867, CST, Danvers, MA), anti-MFN2 (1 : 1000, #9482, CST), anti-OPA1 (1 : 1000, #80471, CST), anti-BAX (1 : 1000, #14796, CST), anti-BCL-2 (1 : 1000, #3498, CST), anti-caspase-3 (1 : 1000, #9661, CST), anti-eIF2*α* (1 : 1000, #5324, CST), anti-p-eIF2*α* (1 : 1000, #3398, CST), anti-GRP78 (1 : 1000, ab21685, Abcam, Cambridge, MA), anti-CHOP (1 : 1000, #2895, CST), anti-COX IV (1 : 1000, ab33985, Abcam), and anti-*β*-actin (1 : 5000, ab8226, Abcam). After several washes, the membranes were incubated with appropriate secondary antibodies, and then, the immunoblots were visualized by enhanced chemiluminescence (Amersham Bioscience, Uppsala, Sweden). Quantification of the bands was performed using ImageJ software (NIH).

### 2.7. ROS Production

ROS levels were detected using an ROS assay kit (Beyotime Biotechnology, Shanghai, China) according to the manufacturer's instructions. The CD4^+^ T cells in each group were incubated with DCFH-DA (10 *μ*M) at 37°C for 20 min and then washed three times. Fluorescence intensity of DCF was measured with a fluorescence microscope (Leica DMi8-M, Germany) at 488 nm (excitation) and 525 nm (emission). The ROS levels were determined according to the fluorescence intensity of DCF.

### 2.8. Transmission Electron Microscopy

Purified cells (4 × 10^6^ cells/mL) were suspended in 2.5% glutaraldehyde and stored at 4°C. First, the samples were fixed with osmic acid in the dark for 1 h after washes. Second, the samples were dehydrated with 50%, 70%, 80%, 90%, and 100% ethanol and 100% acetone after several washes. Third, the samples were infiltrated in a mixture of acetone and embedding medium (1 : 1) for 1 h and then infiltrated in a mixture of acetone and embedding medium (1 : 2) for 3 h. Fourth, the samples were infiltrated in embedding medium overnight. Fifth, the samples were embedded in Eponate. Finally, images were acquired using a JEMI 230 transmission electron microscope, and the areas and lengths of mitochondria were measured by tracing using ImageJ software (NIH).

### 2.9. Statistical Analysis

The data were represented as the mean ± standard deviation (SD) using PSS (version 20.0). A one-way ANOVA was used to analyze significant differences between three or more groups, and unpaired Student's *t*-test was used to analyze significant differences between two groups and the significance was defined as *P* < 0.05. GraphPad Prism 6 (San Diego, CA, USA) was used for the figure design.

## 3. Results

### 3.1. Mdivi-1 Decreases Apoptosis of CD4^+^ T Cells

In the present study, we used CLP surgery to establish an animal septic model *in vivo.* To determine the effect of Mdivi-1 on the mortality of mice and apoptosis of CD4^+^ T cells after the CLP procedure, we pretreated the mice with Mdivi-1 1 h prior to the CLP surgery (Figure S1A). The survival of the mice from each group was recorded for over 7 days (*n* = 54, *P* < 0.05; Figure 2(a)). The CLP mice treated with Mdivi-1 exhibited an increase in the survival rate when compared with the vehicle-treated CLP mice (74.07% vs. 44.44%). We measured the viability and apoptosis of CD4^+^ T cells based upon T cell proliferation the TUNEL assay. As shown in Figure 2(b), the viability of CD4^+^ T cells was significantly decreased at 24 h after CLP surgery whereas Mdivi-1 treatment reversed this decrease. Compared to the sham group, the apoptosis of CD4^+^ T cells was markedly increased in the CLP group (Figure 2(c)), whereas CLP mice subjected to Mdivi-1 treatment significantly reduced apoptosis of CD4^+^ T cells (Figure 2(c)).

Taken together, these data indicate that apoptosis of CD4^+^ T cells induced by CLP was reversed after Mdivi-1 treatment.

### 3.2. Mdivi-1 Corrects the Aberrant Mitochondrial Fusion-Fission Balance

**Figure 5:**
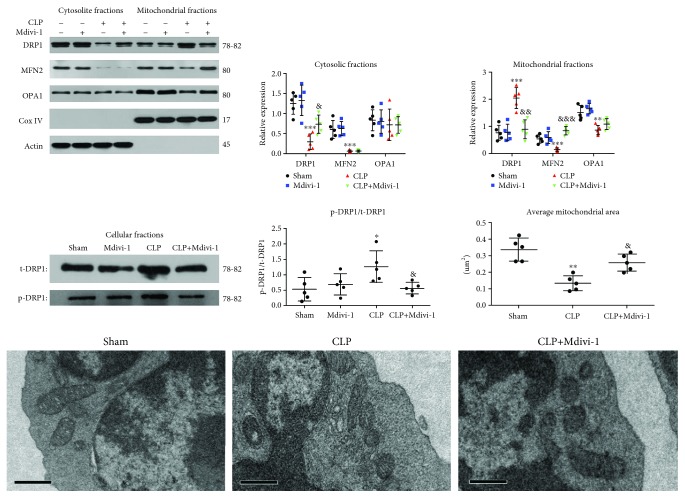
Mdivi-1 reestablished the mitochondrial fusion-fission balance in the CLP mice. Mice were pretreated with Mdivi-1 (20 mg/kg) before undergoing CLP surgery and were sacrificed at 24 h after CLP surgery. (a) Western blot assay and quantitative analysis of mitochondrial and cytosolic MFN2, OPA1, and Drp1 expression. (b) Western blot assay and quantitative analysis of p-Drp1 expression. (c) Micrographs of mitochondria observed using a transmission electron microscopy and quantitative analysis of the average mitochondrial area in five CD4^+^ T cells (magnification: 10000x). Scale bars, 0.5 *μ*m. The results are shown as the mean ± SD; *n* = 5. ^∗^ *P* < 0.05 vs. the sham group (^∗∗^ *P* < 0.01, ^∗∗∗^ *P* < 0.001); ^&^ *P* < 0.05 vs. the CLP group (^&&^ *P* < 0.01, ^&&&^ *P* < 0.001).

The mitochondrial fusion and fission modulates the mitochondrial size and number, and the mitochondrial fusion and fission is balanced in physiological conditions [11]. The mitochondrial fusion-fission balance was evaluated according to the expression levels of MFN2 and OPA1, Drp1 translocation, Drp1 phosphorylation, and mitochondrial morphology, as observed by electron microscopy. Compared to the sham group, mitochondrial MFN2 and OPA1 expression was significantly decreased whereas mitochondrial Drp1 expression was significantly increased in the CLP group (Figure 5(a)). Accordingly, the cytosolic expression of Drp1 was markedly reduced at 24 h post-CLP (Figure 5(a)) whereas the cytosolic expression of MFN2 was decreased and the cytosolic expression of OPA1 was not significantly altered at 24 h post-CLP (Figure 5(a)). Furthermore, the expression of Drp1 and OPA1 in total homogenate was not significantly altered and the expression of MFN2 in total homogenate was decreased at 24 h post-CLP (Figure S1B). Drp1 phosphorylation is correlated with its activity and its recruitment to the mitochondria. Our results showed that Drp1 phosphorylation was increased in the CLP group (Figure 5(b)). To further determine whether mitochondrial morphology was altered in the CD4^+^ T cells, we observed the mitochondrial morphology using a TEM. Our data revealed that the mitochondrial size and mitochondrial length were markedly decreased in the CLP group (Figure 5(c) and Figure S1C).

Next, we determined whether Mdivi-1 treatment affected the mitochondrial dynamics during CLP. Compared to the CLP group, the mitochondrial expression of MFN2 was markedly increased and Drp1 translocation to the mitochondria was markedly decreased after Mdivi-1 treatment (Figure 5(a)). As shown in Figure S1B, the expression of Drp1, MFN2, and OPA1 in total homogenate was not significantly altered after Mdivi-1 treatment. Treatment with Mdivi-1 also decreased Drp1 phosphorylation during CLP (Figure 5(b)). In addition, the mitochondrial size and mitochondrial length were increased after Mdivi-1 treatment compared with the CLP group (Figure 5(c) and Figure S1C). Collectively, these findings demonstrated that Mdivi-1 corrected the aberrant mitochondrial fusion-fission balance.

### 3.3. Mdivi-1 Prevents the Induction of ER Stress

**Figure 6:**
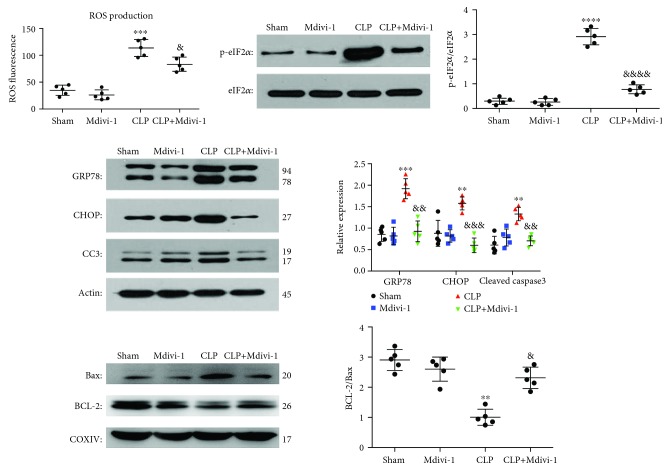
Mdivi-1 reduced ROS production and prevented the induction of ER stress in CD4^+^ T cells after CLP. Mice were sacrificed at 24 h after CLP with or without Mdivi-1 treatment. (a) Quantification of ROS fluorescence intensity. (b) Western blot assay and quantitative analysis of p-eIF2*α* and eIF2*α* expression. (c) Western blot assay and quantitative analysis of GRP78, CHOP, and cleaved caspase-3 expression. (d) Western blot assay and quantitative analysis of BCL-2 and BAX expression. The results are shown as the mean ± SD; *n* = 5. ^∗^ *P* < 0.05 vs. the sham group (^∗∗^ *P* < 0.01, ^∗∗∗^ *P* < 0.001, and ^∗∗∗∗^ *P* < 0.0001); ^&^ *P* < 0.05 vs. the CLP group (^&&^ *P* < 0.01, ^&&&^ *P* < 0.001, and ^&&&&^ *P* < 0.0001).

Increased mitochondrial fission results in excessive ROS release, which can in turn induce ER stress and cell apoptosis [12, 19]. To better understand how Mivid-1 protects CD4^+^ T cells against apoptosis after CLP, we measured the ROS generation and the expression levels of ER chaperones among the different groups. As shown in Figure 6(a), ROS levels were markedly increased at 24 h post-CLP surgery and this increase was reversed by Mdivi-1. Moreover, the levels of the ER stress markers p-eIF2*α* and GRP78 were significantly increased at 24 h post-CLP surgery (Figures 6(b) and 6(c)). Interestingly, Mdivi-1 treatment reversed this CLP-induced increase in p-eIF2*α* and GRP78 expression (Figures 6(b) and 6(c)). Two critical proteins for regulating ERS-related apoptosis CHOP and cleaved caspase-3 were both markedly increased at 24 h post-CLP, and this increase was restored by Mdivi-1 treatment (Figure 6(c)). BAX and BCL-2 have considerable involvement in intrinsic apoptosis through permeabilizing the mitochondrial outer membrane. Compared to the sham group, the BCL-2 : BAX ratio was significantly decreased in the CLP group (Figure 6(d) and Figure S1D), whereas CLP mice subjected to Mdivi-1 treatment increased the BCL-2 : BAX ratio (Figure 6(d) and Figure S1D).

### 3.4. Mdivi-1 Attenuates the LPS-Induced Apoptosis in CD4^+^ T Cells

To further study the impact of Mdivi-1 on the apoptosis of CD4^+^ T cells in sepsis, we used LPS (100 ng/mL, 24 h) stimulation *in vitro* (Figure S2A). We analyzed T cell proliferation and used a TUNEL assay to measure the viability and apoptosis of CD4^+^ T cells after LPS stimulation. First, we activated CD4^+^ T cells via using ConA (5 *μ*g/mL) for 24 h and then measured the expression levels of CD69 in CD4^+^ T cells. We found that CD69 expression was increased after treatment with ConA, indicating that CD4^+^ T cells were activated by ConA (Figure S2B). As shown in Figure 3(b), LPS treatment significantly decreased viability of CD4^+^ T cells. After treatment with Mdivi-1 (10 *μ*M and 20 *μ*M), the LPS-induced decrease in viability of CD4^+^ T cells was reversed (Figure 3(b)). Furthermore, our results also showed that the percentage of TUNEL-positive cells was markedly increased after LPS treatment (Figure 3(a)). In comparison with the LPS+vehicle group, the percentage of TUNEL-positive cells was markedly reduced in the Mdivi-1+LPS group (Figure 3(a)). Therefore, Mdivi-1 attenuated LPS-induced apoptosis in CD4^+^ T cells *in vitro.*

### 3.5. Mdivi-1 Restores the LPS-Induced Abnormal Mitochondrial Fusion-Fission Balance

**Figure 7:**
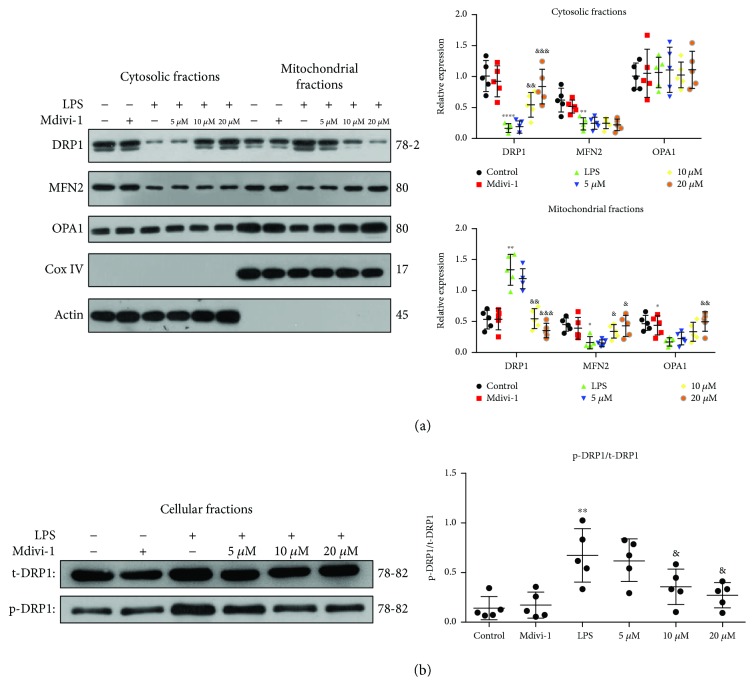
Mdivi-1 reduced Drp1 translocation and Drp1 phosphorylation after the LPS treatment. Mdivi-1 (0, 10, and 20 *μ*M) was administrated to activated CD4^+^ T cells 30 min prior to the LPS stimulation. (a) Western blot assay and quantitative analysis of mitochondrial MFN2, OPA1, and Drp1 expression and cytosolic MFN2, OPA1, and Drp1 expression. (b) Western blot assay and quantitative analysis of Drp1 phosphorylation. The results are shown as the mean ± SD; *n* = 5. ^∗^ *P* < 0.05 vs. the control group (^∗∗^ *P* < 0.01, ^∗∗∗^ *P* < 0.001, and ^∗∗∗∗^ *P* < 0.0001); ^&^ *P* < 0.05 vs. the LPS group (^&&^ *P* < 0.01, ^&&&^ *P* < 0.001).

**Figure 8:**
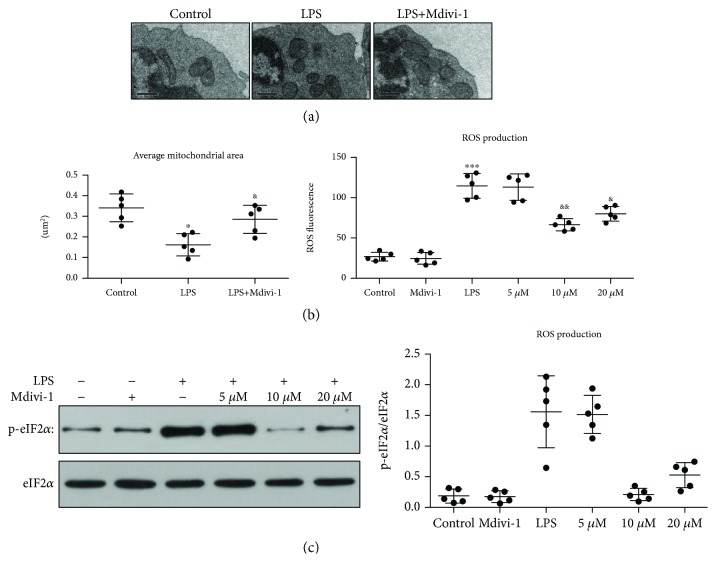
Mdivi-1 restored mitochondrial morphology after LPS treatment. Additionally, Mdivi-1 reduced ROS production and p-eIF2*α* expression after the LPS treatment. (a) Micrographs of mitochondria observed using a transmission electron microscopy and quantitative analysis of the average mitochondrial area in five CD4^+^ T cells (magnification: 10000x). (b) Quantification of ROS fluorescence intensity. (c) Western blot assay and quantitative analysis of p-eIF2*α* and eIF2*α* expression. Scale bars, 0.5 *μ*m. The results are shown as the mean ± SD; *n* = 5. ^∗^ *P* < 0.05 vs. the control group (^∗∗^ *P* < 0.01, ^∗∗∗^ *P* < 0.001); ^&^ *P* < 0.05 vs. the LPS group (^&&^ *P* < 0.01).

First, we measured the expression and activity of the primary GTPase involved in mitochondrial fusion and fission and observed mitochondrial morphology at 24 h after the LPS treatment. Our findings showed that the mitochondrial MFN2 and OPA1 expression was decreased whereas mitochondrial Drp1 expression was markedly increased after the LPS treatment (Figure 7(a)). Accordingly, cytosolic Drp1 was significantly decreased after the LPS treatment which indicates that Drp1 translocation to the mitochondria was increased (Figure 7(a)). Interestingly, cytosolic MFN2 was decreased and cytosolic OPA1 was not significantly altered after the LPS treatment (Figure 7(a)). We also found that Drp1 phosphorylation was markedly increased after the LPS treatment (Figure 7(b)). Furthermore, our data showed that the mitochondrial size and mitochondrial length were decreased after the LPS treatment (Figure 8(a) and Figure S2C)

Next, we determined whether various concentrations of Mdivi-1 affected mitochondrial morphology and the expression and activity of the main GTPase after LPS treatment. Compared to the LPS group, the mitochondrial MFN2 and OPA1 expression was increased and mitochondrial Drp1 expression and Drp1 phosphorylation were decreased after treatment with Mdivi-1 (10 *μ*M and 20 *μ*M) (Figures 7(a) and 7(b)). Accordingly, 10 *μ*M and 20 *μ*M Mdivi-1 significantly increased the cytosolic Drp1 expression after LPS treatment (Figure 7(a)). Our findings indicate that Mdivi-1 treatment decreases the translocation of Drp1 and Drp1 activity after LPS treatment. Moreover, Mdivi-1 increased the mitochondrial size and mitochondrial length after LPS treatment (Figure 8(a) and Figure S2C).

### 3.6. Mdivi-1 Decreased the Release of ROS and Prevented the Induction of LPS-Induced ER Stress

**Figure 9:**
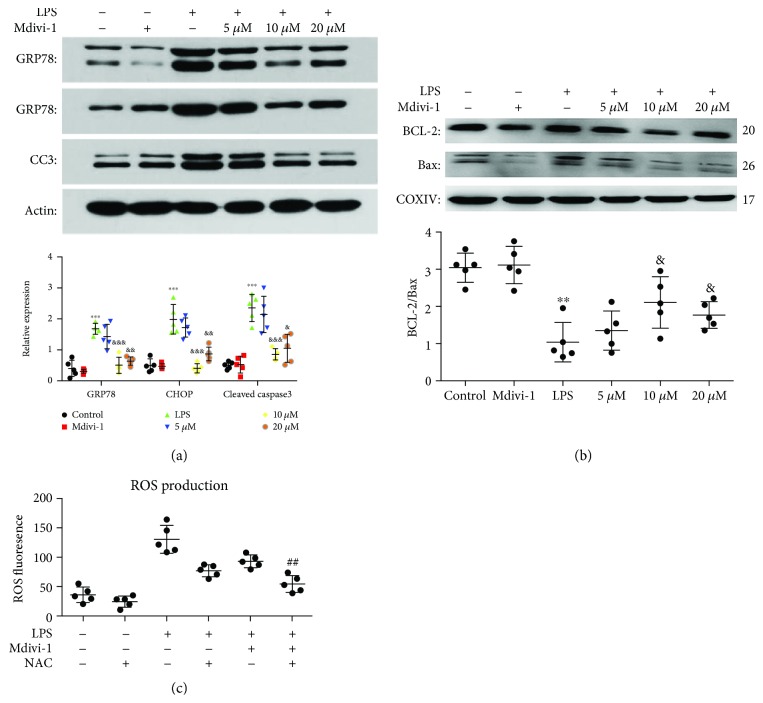
Mdivi-1 prevented the induction of ER stress during sepsis. Additionally, ROS levels were reduced after NAC treatment. (a) Western blot assay and quantitative analysis of GRP78, CHOP, and cleaved caspase-3 expression. (b) Western blot assay and quantitative analysis of BCL-2 and BAX expression. (c) Quantification of ROS fluorescence intensity. The results are shown as the mean ± SD; *n* = 5. ^∗∗^ *P* < 0.01 vs. the control group (^∗∗∗^ *P* < 0.001); ^&^ *P* < 0.05 vs. the LPS group (^&&^ *P* < 0.01, ^&&&^ *P* < 0.001); ^##^ *P* < 0.01 vs. the LPS+Mdivi-1 group.

Our previous studies demonstrated that the induction of ER stress is increased in dendritic cells after severe thermal injury and the inhibition of ER stress reduces the dysfunction and apoptosis of dendritic cells [24, 25]. First, we assessed the levels of ROS and the expression of ER chaperones at 24 h after the LPS treatment. ROS levels and the p-eIF2*α*, GRP78, CHOP and cleaved caspase-3 expression were increased, and the ratio of BCL-2 : BAX was decreased after LPS treatment (Figures 8(b), 8(c), 9(a), and 9(b) and Figure S3A). These findings demonstrate that the release of ROS was increased, and ER stress was induced at 24 h after the LPS treatment.

Next, we evaluated whether Mdivi-1 affects the release of ROS and the induction of ER stress after LPS treatment. Treatment with 10 *μ*M and 20 *μ*M Mdivi-1 reduced the release of ROS and the p-eIF2*α*, GRP78, CHOP, and caspase-3 expression and increased the BCL-2 : BAX ratio after LPS treatment (Figures 8(b), 8(c), 9(a), and 9(b) and Figure S3A).

### 3.7. Prevention of ROS Production Amplified Mdivi-1-Induced Prevention of ER Stress

**Figure 10:**
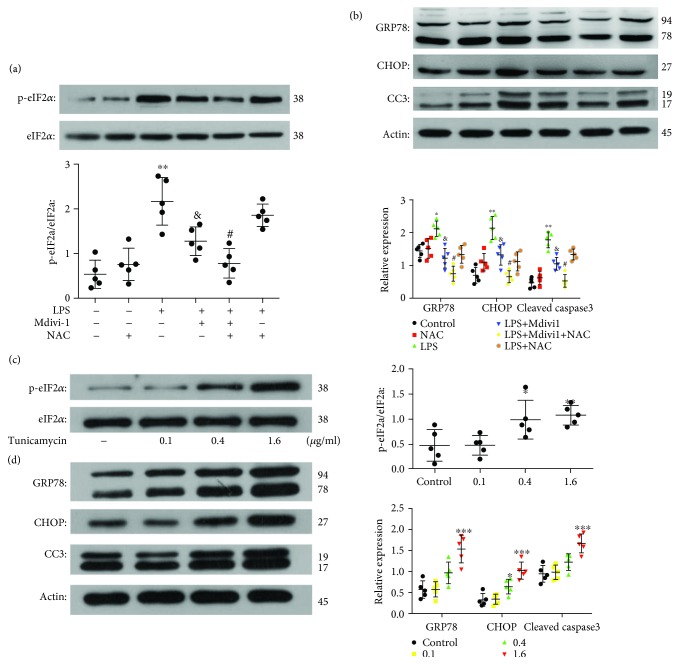
NAC treatment further reduced the induction of ER stress after cotreatment with LPS and Mdivi-1. Moreover, ER stress was induced after tunicamycin treatment. (a, c) Western blot assay and quantitative analysis of p-eIF2*α* and eIF2*α* expression. (b, d) Western blot assay and quantitative analysis of GRP78, CHOP, and cleaved caspase-3 expression. The results are shown as the mean ± SD; *n* = 5. ^∗^ *P* < 0.05) vs. the control group (^∗∗^ *P* < 0.01,^∗∗∗^ *P* < 0.001); ^&^ *P* < 0.05 vs. the LPS group; ^#^ *P* < 0.05 vs. the LPS+Mdivi-1 group (*P* < 0.05). ^#^Statistically significant difference compared with the LPS+Mdivi-1 group (*P* < 0.05)

To explore a potential connection between mitochondrial dynamics and ER stress after Mdivi-1 administration, we measured the expression of ER chaperones after the prevention of ROS release (Figure S3B). As shown in Figure 9(c), ROS release was prevented by NAC. Moreover, the prevention of ROS release further decreased the p-eIF2*α*, GRP78, CHOP, and caspase-3 expression after the Mdivi-1 treatment (Figures 10(a) and 10(b)). These data suggested that Mdivi-1 prevented induction of ER stress by, at least partly, decreasing ROS production.

### 3.8. ERS Induction Compromised and ERS Prevention Amplified the Protective Effects of Mdivi-1 on CD4^+^ T Cells after LPS Treatment

To further study the potential mechanism underlying the protective effects of Mdivi-1 on CD4^+^ T cells, we administered tunicamycin to induce ER stress and 4-PBA to alleviate ER stress (Figure S3B). The results showed that tunicamycin (1.6 *μ*g/mL) robustly increased the ER stress markers p-eIF2*α* and GRP78 and two factors in ERS-related apoptosis, CHOP, and cleaved caspase-3 (Figures 10(c) and 10(d)). In contrast, 4-PBA (2 mmol/L) decreased the expression of GRP78 and cleaved caspase-3 after LPS treatment (Figure S3C). To confirm whether ERS induction and ERS prevention affect the antiapoptosis effect of Mdivi-1 on CD4^+^ T cells after LPS treatment, we detected the viability and apoptosis of CD4^+^ T cells by T cell proliferation and TUNEL assay among the different groups. Apoptosis of CD4^+^ T cells was significantly increased in the LPS+Mdivi-1+tunicamycin group when compared with the LPS+Mdivi-1 group (Figure 4(a)). Apoptosis of CD4^+^ T cells was significantly decreased in the LPS+Mdivi-1+4-PBA group when compared with the LPS+Mdivi-1 group (Figure 4(a)). Compared to the LPS+Mdivi-1 group, the viability of CD4^+^ T cells was decreased in the LPS+Mdivi-1+tunicamycin group and the viability of CD4^+^ T cells had no significant changes in the LPS+Mdivi-1+4-PBA group (Figure 4(b)). These data suggested that Mdivi-1 protected CD4^+^ T cells against apoptosis through, at least partly, preventing the induction of ER stress.

## 4. Discussion

Sepsis is a leading cause of ICU death and is characterized by an uncontrolled inflammatory response in the initial phase and immunosuppression in the late phase [26, 27]. Despite therapeutic advances, including immunotherapy, early fluid resuscitation, and organ system support, sepsis still has a poor outcome for patients because the pathology of sepsis remains unclear [28, 29]. Following a period of hyperinflammatory response, a compensatory anti-inflammatory response and even immune suppression occur, which are the main causes of high mortality in the late phase of sepsis [4]. Recent studies and clinical findings have demonstrated that apoptosis of T lymphocytes has a considerable involvement in immunosuppression and is critically related to the outcome of sepsis [30–32]. Here, our study showed that apoptosis in CD4^+^ T cells was increased after LPS administration and CLP surgery. Consequently, it is urgent to develop novel therapeutic strategies to attenuate apoptosis in CD4^+^ T cells during sepsis to affect the outcome.

Midvi-1, which is a derivative of quinazolinone, has been reported to be an inhibitor of Drp1 function [17]. Several studies have demonstrated targets of Mdivi-1 in numerous diseases that range from diseases of the nervous system to heart disease [9, 33], because Mdivi-1 reduces cell injury and apoptosis. However, recent studies have also shown that Mdivi-1 suppresses mitophagy, resulting in aggravating neuronal injury [34, 35]. In our study, our data demonstrated that Mdivi-1 increased the survival rate of CLP mice and reduced apoptosis of CD4^+^ T cells in experimental sepsis. To explore the mechanisms underlying the protective effects of Mdivi-1, we speculated that Mdivi-1 reduced apoptosis of CD4^+^ T cells by balancing mitochondrial fusion-fission and preventing the induction of ER stress.

Mitochondrial quality control mechanisms eliminate damaged and aged mitochondria to maintain a pool of healthy mitochondria through a series of processes, including mitochondrial biogenesis, mitochondrial dynamics, and mitophagy [36]. Mitochondrial dynamics are regulated by evolutionarily conserved dynamin-related GTPases, including the fission protein Drp1; its receptors Fis1, MFF, MiD49, and MiD51; and the fusion proteins Mfn1, MFN2, and Opa1, to modulate mitochondrial morphology, number and size [15]. Therefore, a balance among these dynamic processes plays a central role on counteracting deleterious mitochondrial processes and maintaining normal cellular function. In pathologic states, morphological analysis reveals that the long tubular-shaped mitochondria are shortened and mitochondrial fragmentation is increased, which are characteristics of mitochondrial fission [37]. Similarly, our results showed that the mitochondrial fusion-fission balance shifts toward mitochondrial fission during sepsis, as reflected by a decrease in MFN2 and OPA1, an increase in Drp1 translocation and Drp1 phosphorylation, and a decrease in mitochondrial size and elongated mitochondria. An increase in mitochondrial fragmentation which disrupts normal mitochondrial functions results in excessive ROS release and induction of ER stress [38]. After Mdivi-1 administration, the mitochondrial expression of MFN2 and OPA1 was increased, Drp1 translocation and Drp1 phosphorylation were decreased, and the mitochondrial size and elongated mitochondria were increased. Consequently, Mdivi-1 restores the mitochondrial fusion-fission balance in sepsis. The underlying molecular mechanism may be that Mdivi-1 prevents the self-assembly of Drp1, which is required for mitochondrial fission, and the selective inhibition of mitochondrial fission in turn suppresses mitochondrial fusion defects [16, 39].

In the progression of sepsis, the balance of ROS generation and antioxidant defenses is disrupted, resulting in the excessive production of ROS [29]. Furthermore, increased mitochondrial fragmentation also increases the release of ROS. Our results showed that ROS release is significantly increased during sepsis. High levels of ROS generation partially affect ER homeostasis, and then, unfolded proteins are accumulated in the ER [18, 40]. In response to ER stress, three signaling pathways are activated, which trigger several transduction factors that determine cell survival or apoptosis. Cell apoptosis results from acute or chronic induction of ER stress [19, 41]. The proapoptotic factor BAX is recruited to the mitochondrial outer membrane during chronic induction of ER stress, which in turn permeabilizes the mitochondrial outer membrane and increases the release of cytochrome *c* [42, 43]. Caspase-9, an activator of caspase-3, is activated by cytochrome *c*, which finally induces cell apoptosis [44]. In our study, we indicate that the expression levels of the ER stress markers p-eIF2*α* and GRP78 and two factors involved in ERS-related apoptosis, CHOP and Caspase-3, were increased, and the BCL-2 : BAX ratio was decreased during sepsis. After treatment with Mdivi-1, ROS production was reduced and the induction of ER stress was prevented. Our results indicate that the Mdivi-1-induced decrease in mitochondrial fragmentation reduced ROS production and prevented ER stress in sepsis. To further determine whether ROS production represents a connection between mitochondrial dynamics and ER stress, we inhibited ROS production using NAC. Our data showed that p-eIF2*α*, GRP78, CHOP, and CC3 expression was decreased after NAC treatment, indicating that ROS is a mediator of ER stress.

Additionally, to further explore whether preventing ER stress is a mechanism involved in the protective role of Mdivi-1 on CD4^+^ T cells in sepsis, we used tunicamycin to induce ER stress and 4-PBA to alleviate ER stress. Tunicamycin induced ER stress and eliminated the protective effect of Mdivi-1 treatment on CD4^+^ T cells during sepsis. In contrast, 4-PBA decreased the expression of GRP78 and cleaved caspase-3 and amplified the protective effect of Mdivi-1 treatment on CD4^+^ T cells during sepsis. Collectively, we concluded that Mdivi-1 attenuated apoptosis in CD4^+^ T cells through preventing ER stress.

Several limitations in this study warrant further investigation. First, it is known that three signal transducers, including IRE1, PERK, and ATF6, are activated in response to ERS, which then activate several transcription factors to cope with the stress. In a future study, the precise signaling pathway through which Mdivi-1 reduces apoptosis in CD4^+^ T cells should be investigated in the setting of a septic challenge. Second, it is important to determine whether Mdivi-1 inhibits mitophagy and, if so, whether such inhibition of mitophagy by Mdivi-1 affects cell apoptosis.

In summary, the results of the present study suggest that Mdivi-1 reduces apoptosis in CD4^+^ T cells during sepsis. Balancing mitochondrial fusion-fission and preventing ER stress are two potential mechanisms involved in the protective effect of Mdivi-1 on CD4^+^ T cells in sepsis.

## 5. Conclusion

To our knowledge, whether Mdivi-1 affects apoptosis of immune cells has not been elucidated so far. In our manuscript, we demonstrated that Mdivi-1 reduces apoptosis in CD4^+^ T cells during sepsis and balancing mitochondrial fusion-fission and preventing ER stress are two potential mechanisms involved in the protective effect of Mdivi-1 on CD4^+^ T cells in sepsis. Therefore, Mdivi-1 treatment as a novel therapeutic strategy targeted apoptosis of CD4^+^ T cells during sepsis.

## Data Availability

The data used to support the finding of this study are available from the corresponding authors upon request.

## Supplementary Materials

Supplementary Materials Figure S1: (A) the experimental protocol is related to Figure 2, and the schematic panel illustrates the study design. (B) Western blot assay of MFN2, OPA1 p-Drp1, and Drp1 expression in total homogenate. (C) Micrographs of mitochondria observed using a transmission electron microscopy in Figure 5(c) and quantitative analysis of average mitochondrial length in five CD4^+^ T cells. (D) Quantitative analysis of BCL-2 and Bax. The results are shown as the mean ± SD; *n* = 5. ^∗^ *P* < 0.05 vs. the sham group (^∗∗∗^ *P* < 0.001); ^&^ *P* < 0.05 vs. the CLP group (^&&^ *P* < 0.01). Figure S2: (A) the experimental protocol is related to Figure 3, and the schematic panel illustrates the study design. (B) Expression levels of CD4 and CD69 by flow cytometric analysis in CD4^+^ T cells. (C) Micrographs of mitochondria observed using a transmission electron microscopy in Figure 8(a) and quantitative analysis of average mitochondrial length in five CD4^+^ T cells. The results are shown as the mean ± SD; *n* = 5. ^∗∗∗^ *P* < 0.001 vs. the sham group; ^&&^ *P* < 0.01 vs. the CLP group. Figure S3: (A) quantitative analysis of BCL-2 and Bax. (B) The experimental protocol is related to Figures 10 and 4, and the schematic panel illustrates the study design. (C) Western blot assay and quantitative analysis of GRP78 and CC3 expression. The results are shown as the mean ± SD; *n* = 5. ^∗^ *P* < 0.05 vs. the control group (^∗∗∗^ *P* < 0.001); ^&^ *P* < 0.05 vs. the CLP group (^&&^ *P* < 0.01, ^&&&^ *P* < 0.001).
