# Supplementary material for: Mdivi-1 Protects CD4+ T Cells against Apoptosis via Balancing Mitochondrial Fusion-Fission and Preventing the Induction of Endoplasmic Reticulum Stress in Sepsis
Source: Mediators Inflamm. 2019 May 16;2019:7329131. doi: 10.1155/2019/7329131 (PMC6541989; doi:10.1155/2019/7329131)
Supplement: Supplementary Materials — Figure S1: (A) the experimental protocol is related to Figure 2, and the schematic panel illustrates the study design. (B) Western blot assay of MFN2, OPA1 p-Drp1, and Drp1 expression in total homogenate. (C) Micrographs of mitochondria observed using a transmission electron microscopy in Figure 5(c) and quantitative analysis of average mitochondrial length in five CD4+ T cells. (D) Quantitative analysis of BCL-2 and Bax. The results are shown as the mean ± SD; n = 5. ∗ P < 0.05 vs. the sham group (∗∗∗ P < 0.001); & P < 0.05 vs. the CLP group (&& P < 0.01). Figure S2: (A) the experimental protocol is related to Figure 3, and the schematic panel illustrates the study design. (B) Expression levels of CD4 and CD69 by flow cytometric analysis in CD4+ T cells. (C) Micrographs of mitochondria observed using a transmission electron microscopy in Figure 8(a) and quantitative analysis of average mitochondrial length in five CD4+ T cells. The results are shown as the mean ± SD; n = 5. ∗∗∗ P < 0.001 vs. the sham group; && P < 0.01 vs. the CLP group. Figure S3: (A) quantitative analysis of BCL-2 and Bax. (B) The experimental protocol is related to Figures 10 and 4, and the schematic panel illustrates the study design. (C) Western blot assay and quantitative analysis of GRP78 and CC3 expression. The results are shown as the mean ± SD; n = 5. ∗ P < 0.05 vs. the control group (∗∗∗ P < 0.001); & P < 0.05 vs. the CLP group (&& P < 0.01, &&& P < 0.001). [file 7329131.f1.pdf]

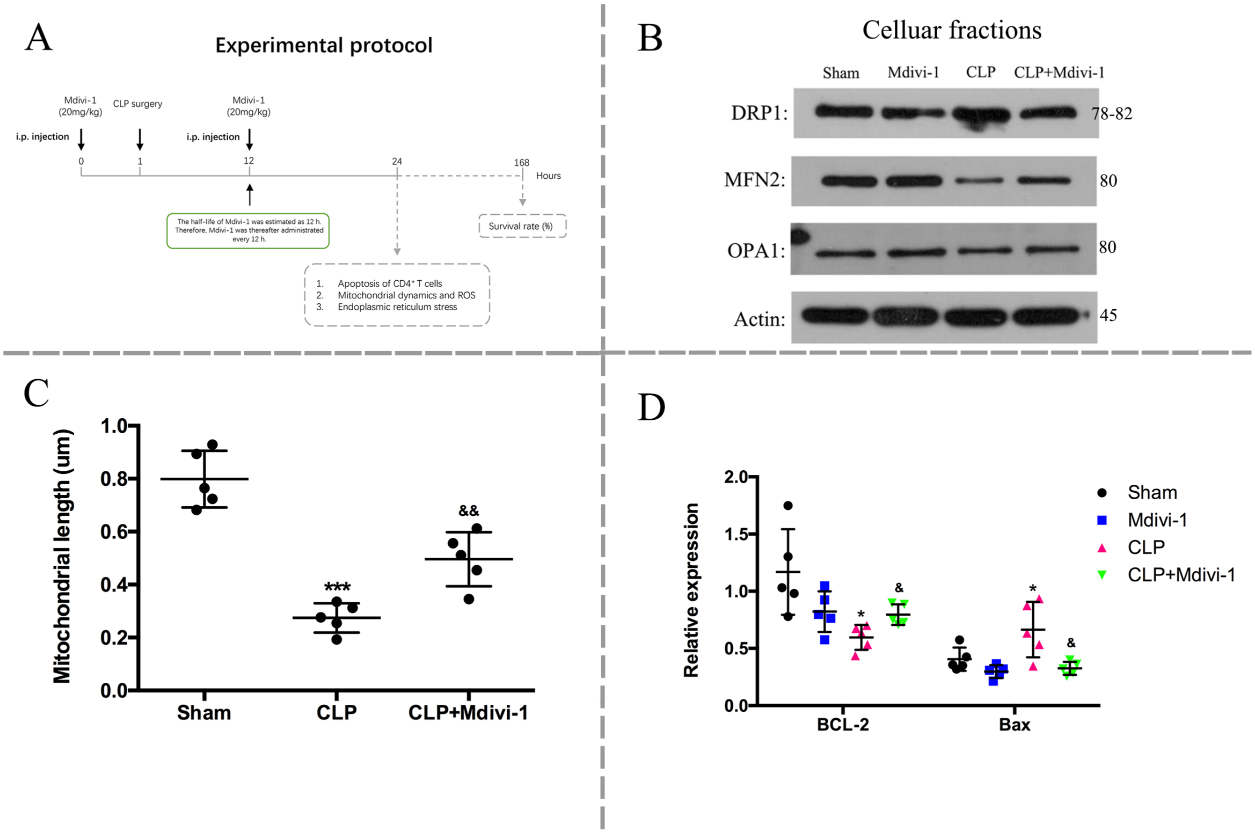


**Figure S1.** (A) The experimental protocol is related to Figure 1 and schematic panel illustrates the study design; (B) Western blot assay of MFN2, OPA1 p-Drp1 and Drp1 expression in total homogenate; (C) Micrographs of mitochondria observed using a transmission electron microscopy in Figure 2C and quantitative analysis of average mitochondrial length in five $CD4^+$T cells; (D) quantitative analysis of BCL-2 and Bax. The results are shown as the mean ± SD, n=5. *(P< 0.05) vs the sham group, ***(P< 0.001); $^{\&}$(P< 0.05) vs the CLP group, $^{\&\&}$(P< 0.01).

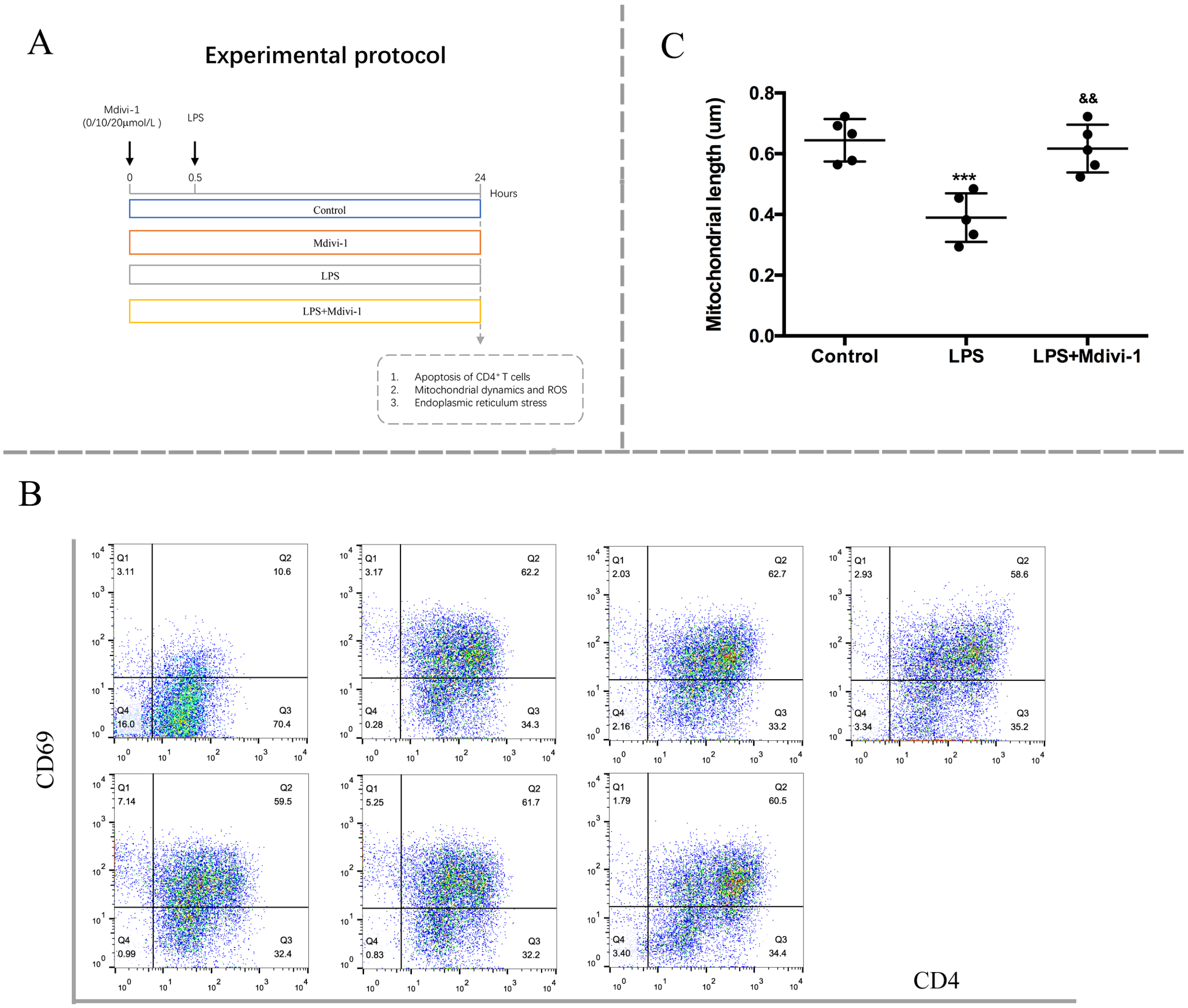


**Figure S2.** (A)The experimental protocol is related to Figure 4 and schematic panel illustrates the study design; (B) Expression levels of CD4 and CD69 by flow cytometric analysis in CD4$^+$T cells; (C) Micrographs of mitochondria observed using a transmission electron microscopy in Figure 6A and quantitative analysis of average mitochondrial length in five CD4$^+$T cells. The results are shown as the mean ± SD, n=5. ***(P< 0.001) vs the sham group; $^{\&\&}$(P< 0.01) vs the CLP group.

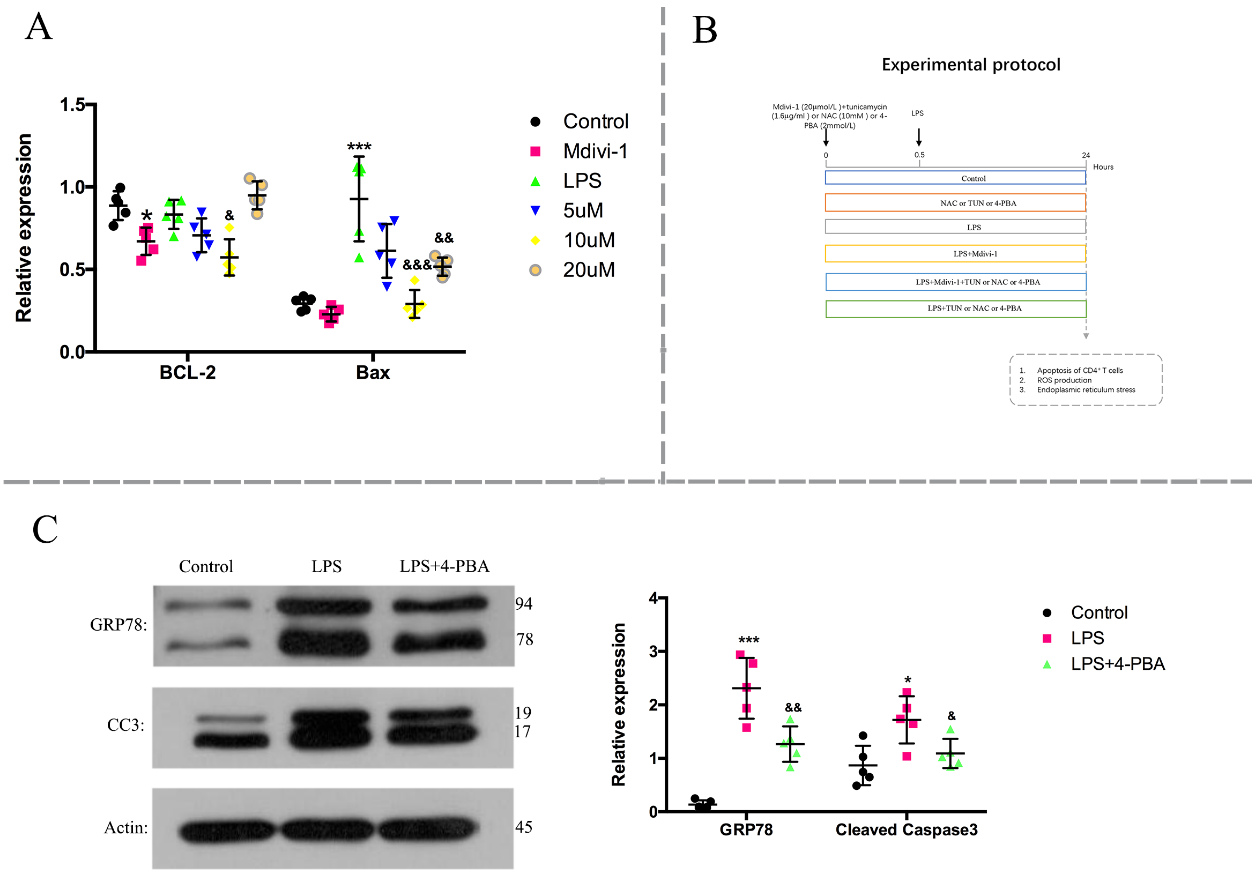


**Figure S3.** (A) quantitative analysis of BCL-2 and Bax; (B) The experimental protocol is related to Figure 8, 9 and schematic panel illustrates the study design; (C) Western blot assay and quantitative analysis of GRP78 and CC3 expression. The results are shown as the mean ± SD, n=5. *(P< 0.05) vs the Control group, ***(P< 0.001); $^{\&}$(P< 0.05) vs the CLP group, $^{\&\&}$(P< 0.01), $^{\&\&\&}$ (P< 0.001).
